# Perceived conflict in the couple and chronic illness management: Preliminary analyses from the Quebec Health Survey

**DOI:** 10.1186/1471-2296-7-59

**Published:** 2006-10-19

**Authors:** Hassan Soubhi, Martin Fortin, Catherine Hudon

**Affiliations:** 1 Department of Family Medicine, Sherbrooke University, Sherbrooke, Que, Canada, Unité de médecine de famille, 305 St-Vallier, Chicoutimi, Qc, G7H 5H6, Canada

## Abstract

**Background:**

The quality of the relationship with the spouse/partner appears crucial among patients with multiple chronic conditions where illness management is complex and multifaceted. This study draws on data from the Quebec Health Survey (QHS) to examine, among patients with one or more chronic conditions, the relation between marital status, the perceived conflict with the spouse/partner, and what the patients do to manage their illness as well as how they perceive their health.

**Methods:**

Data from the QHS 1998 were used. The sample included 7547 coupled adults who had one or more chronic health problems lasting more than 6 months. Independent variables included marital status, perceived conflict with the spouse/partner, and the number of chronic conditions. Illness management was defined broadly as a measure of the patient's efforts at self-care and an illness status indicator, including visits to the generalist and the specialist, the use of telephone health line in the last 12 months, self-rated general health, mental health, and a measure of psychological distress. Linkages between the independent variables and illness management were assessed for males and females separately with logistic regressions, while accounting for the survey sampling design and household clustering.

**Results:**

Female patients who did not live with their partner and had never been married were more likely to report a negative perception of their general health and a higher psychological distress than those who were married. Perceived conflict with the partner was linked to a negative perception of mental health and a higher psychological distress among both men and women. Compared to patients with only one chronic condition, males who reported more than one chronic condition were more likely to have consulted a generalist prior to the survey and used the telephone health line, whereas females were more likely to have consulted a specialist. Both males and females with more than one chronic condition were more likely to have a negative perception of their general health and mental health.

**Conclusion:**

The study provides a useful preliminary measure of the importance of living arrangements and the quality of the couple relationship in chronic illness management broadly conceived as a measure of the patient's efforts at self-care and an illness status indicator. Results of this study prod us to examine more closely, within longitudinal designs, the influence of living arrangements and the presence of conflict in the couple on chronic illness management as well as the modifying effect of gender on these associations.

## Background

Research indicates that the structure and quality of the couple relationship can influence what chronically ill patients do to manage their illness as well as how they perceive their physical, mental and emotional health [1-7]. Since the treatment of most chronic illnesses includes lifestyle and role changes that have the potential to strain the patient's couple relationship [5], the structure and quality of the relationship with the spouse or partner appear crucial, especially among patients with multiple chronic conditions where illness management is complex and multifaceted [5,6,8,9]. Our purpose in this study is to draw on data from the Quebec Health Survey to examine, among patients with one or more chronic conditions, the relation between marital status, the presence of conflict in the relationship with the spouse/partner, what the patients do to manage their illness as well as how they perceive their health.

### Family context and chronic illness management

Family is a primary source of influence in sustaining health and chronic illness management [1,5,10,11]. Family resources for responding to chronic illness demands include family structure which sets the pattern of roles and rules that guide relationships in the family and that can affect the work that can be accomplished within and outside the family [4,6,12]. Marriage is an example of a socially defined structure of roles and rules that has long been linked with health and illness [13,14]. Married adults have a lower prevalence of illness and are more likely to recover quickly than unmarried adults [4,15]. Marriage may reflect a strong commitment to the relationship and sustained provision of support in the management of illness through greater family organization and sharing of tasks. By contrast, lack of sharing and organization can have an effect on the patient's physiological and quality of life outcomes when they lead the patient to discouragement and isolation [5,6,11].

Another family resource for responding to chronic illness is the family's expression and management of emotion including conflict resolution, intimacy, anger, loss [5,16-18]. A hostile or conflicted family environment may impede the patient's ability to maintain thoughtful daily self-care practices [6]. Family relations marked by conflict or hostility may have a direct, negative effect on the patient's physiology, endocrine balance and subsequent humoral control [6,19]. By contrast, intimate and caring relations can positively influence patient's health by providing emotional support and respite in dealing with disease demands and downturns [1,3].

### The Present Study

The present study builds on previous research in four ways. First, given the multifaceted nature of chronic illness, we adopt a broad view of illness management as a measure of the patient's efforts at self-care and an illness status indicator [6,20]. Work by Chesla et al. [6] and Fisher et al. [20] indicate that the management of chronic illness is most meaningfully assessed across its multiple behavioral and clinical dimensions. Drawing on data from the Quebec Health Survey, we then focus in this study on the visits to the generalist and specialist, the use of the Quebec telephone health line Info-Santé, and the patients' self-rated levels of general health, mental health, and psychological distress. Second, we conceive of these variables as a system of interdependent variables that is rooted within the social context of the patient's couple relationships, and as such can be linked to the structural and functional qualities of these relationships [21,22]. Characteristics of the couple relationship can then be thought of as resources or obstacles that can assist or hinder the patient's efforts to live with the demands of illness [2,5]. Third, we extend the investigation of these linkages to patients with multiple chronic conditions – an extension which to our knowledge has not been done for patients in this vulnerable segment of chronically ill patients. Fourth, because of gender differences in self-care, and in the way males and females perceive their couple environment, we analyze the data separately for males and females [19,23].

## Methods

### Data source

Data from the Quebec Health Survey 1998(QHS) were used. Detailed descriptions of the QHS methods and variables are available elsewhere [24]. Briefly, the survey is a multistage random probability sample of Quebec residential households with the goal of collecting data on the health and well being of the Quebec population, the needs and priority areas for intervention, and the allocation of resources [24]. In all, 15 330 households were surveyed. Information about the members of the household was obtained from the person most knowledgeable (PMK) of the health of family members. The survey included two parts conducted in a cascade fashion, one administered by an interviewer and another self-administered. The response rate to the interviewer survey was 82.1%. The response rate to the self-administered survey, tied to the response rate of the interviewer survey, was 69%.

### Subjects

For the purposes of this study, we included respondents who met the following criteria: they were aged 18 years and older at the time of the survey; they declared having a spouse or partner (husband, wife, boyfriend, girlfriend), and one or more chronic health problems lasting more than 6 months. In total 7547 individuals met these inclusion criteria. Table 1 presents the sample characteristics separately for each gender. About 58% of the participants were females, slightly younger than their male counterparts, and about 60% declared having more than one chronic health problem compared to 50% among males.

**Table 1:** Sample socio-demographic Characteristics

**Variables**	**Males (n = 3170)**	**Females (n = 4377)**
**Age**		
18–44	45.4	54.4
45–64	38.5	36.1
65+	16.1	9.5
		
**Education**		
Primary	17.6	12.5
Secondary	37.8	40.0
Diploma/some university	40.6	45.1
University Degree	4.0	2.4
		
**Employment**		
Part/Fulltime	70.0	55.2
Not working	30.0	44.8
		
**Income**		
< 6000$	6.0	31.1
6000–19999	25.2	33.3
20000–39999	38.3	24.5
≥ 40000	30.5	11.1
		
**Marital Status**		
Married*	69.1	66.1
Common Law**	24.6	24.3
Wid/Sep/Div. and do not live with their partner	1.4	2.6
Never married and do not live with their partner	5.0	7.0
		
**Couple Conflict**		
None	74.0	70.2
Little	13.4	12.8
Moderate	7.3	8.6
Severe	5.3	8.4
		
**Number of Chronic Health Problems**		
One	49.8	40.4
More than one	50.2	59.6

* married and living with their spouse; ** living with a partner and married/separated or widowed, or divorced, or never married

### Independent Variables

The set of independent variables included marital status, the level of conflict with the spouse/partner, and the number of chronic health problems. ***Marital Status ***was a categorical variable that indicated the respondents' legal marital status as well as whether they lived with their partner in the same household or not. Four categories were used: 1) Married: married and living with their spouse; 2) Common Law: living with a partner and married/separated or widowed, or divorced, or never married; 3) Widowed or separated or divorced, and not living with their partner; 4) Never married and not living with their partner. ***Conflict with the spouse/partner ***was a categorical variable that assessed the respondent's perceived conflict in the relationship with the spouse/partner. Items included:''the spouse/partner doesn't understand you", ''spouse/partner doesn't show enough affection", and ''spouse/partner is not involved enough in your relationship". Responses were coded as True 1 or False 0. From the summed responses to these items, 4 categories were derived reflecting the perceived level of conflict experienced in the relationship so that the higher the score, the higher the level of conflict in the relationship starting from no conflict 0, little conflict 1, moderate conflict 2, to severe conflict 3. The items used for this scale were derived from work by Turner and Wheaton [25] and the scale shows good internal consistency (Cronbach's alpha = 0.92). ***Number of Chronic Health Problems ***was a dichotomous variable that assessed the presence of one or more chronic health problems lasting more than 6 months. All independent variables were used as dummy variables with the first category as reference category. Finally, socio-demographic characteristics – *age, gender, education, employment, and income *– that may account for part of the variance in illness management were included as covariates in the analyses.

### Dependent Variables

Illness management was conceptualized broadly as the patient's efforts at self-care and an illness status indicator [6]. The patient's efforts at self-care included the use of health services: consulting a generalist, a specialist, and using the telephone health line. The patient's illness status included self-rated general and mental health, and the perceived psychological strain assessed by a measure of psychological distress.

1. *Consulting a generalist and consulting a specialist *were two variables that assessed whether a consultation with a Generalist (No/Yes) or a Specialist (No/Yes) occurred in the last two weeks prior to the survey.

2. *Use of Info-Sant*é assessed the use of the Quebec telephone health information service Info-Santé during the last 12 months preceding the survey. Response choices were No/Yes.

3. *Self-Rated General Health *provided a global assessment of the patient's perception of his/her health status overall compared to others of the same age. Responses were grouped in two categories: Excellent/Good = 0, and Average/Poor = 1

4. *Self-Rated Mental Health *provided a global assessment of the patient's perception of his/her mental health. Responses were grouped into two categories: Excellent/Good = 0, and Average/Poor = 1

5. *Psychological Distress *was a modified version of the Psychiatric Symptom Index of Ilfeld [26]. A high score indicated a greater level of reported psychological distress. The scores were grouped into two categories Low/Average = 0, and High = 1.

### Statistical analyses

Assessment of the associations between the independent variables and each of the dependent variables was done with logistic regressions [27]. To account for the complex sampling design of the survey as well as for household clustering, all analyses were conducted using SUDAAN software version 9.0. Regression parameters were then estimated using GEE with robust variance estimation. Six logistic regression equations (one for each dependent variable) were used to assess the link between the dependent variables and the independent variables (marital status, level of conflict in the relationship with the spouse/partner, and number of chronic health problems). Analyses were conducted separately for males and females. All the covariates (age, education, employment, and income) were the first to be introduced in the logistic equations and were maintained as control variables in all the models. After the main effects, interaction terms were also tested between marital status and the perceived conflict with the spouse/partner, between marital status and the number of chronic health problems, and between the perceived conflict and the number of chronic health problems. Assessment of the fit of the models was based on the Hosmer-Lemeshow Satterwhaite F index with non-significant values indicating a good fit. To account for multiple comparisons, the alpha level of significance was set to .01. Results are expressed as odds ratios with 99% confidence intervals.

## Results

None of the interactions that we tested achieved significance in this study. In the following sections, we present the models without the interaction terms. As indicated in Table 2, for males, marital status did not show any statistically significant pattern of association with any of the dependent variables. Compared to male patients who reported no conflict with their spouse/partner, patients who reported any level of conflict were more likely to describe as average to poor their mental health, and were more likely to report higher levels of psychological distress. Finally, male patients who reported more than one chronic condition were about twice as likely to have consulted a generalist prior to the survey compared to patients with only one chronic condition. They were also more likely to have used info-santé, to report a negative perception of their general health and mental health, and a higher psychological distress.

**Table 2:** Male Patients. Odds ratios and 99% confidence intervals^† ^from logistic regression models^†† ^linking marital status, the perceived conflict with the spouse/partner, and the number of chronic health problems, to chronic illness management variables.

	**Dependent Variables**					
	
**Independent Variables**	Consulting a GP	Consulting a Specialist	Use of Info-Santé	General Health	Mental Health	Psychological Distress
***Marital Status***						
Married*	Ref.					
Common Law**	1.27 (0.74–2.17)	0.84 (0.41–1.74)	1.13 (0.79–1.63)	0.78 (0.46–1.32)	1.07 (0.58–1.97)	1.41 (0.96–2.07)
Wid/Div/Sep and do not live with their partner	0.92 (0.18–4.70)	0.30 (0.04–2.12)	1.02 (0.22–4.74)	0.68 (0.17–2.80)	0.86 (0.16–4.65)	0.77 (0.24–2.50)
Never married and do not live with their partner	0.84 (0.28–2.52)	0.76 (0.18–3.17)	0.78 (0.36–1.67)	0.82 (0.26–2.59)	0.57 (0.17–1.91)	1.28 (0.57–2.87)
						
***Conflict with spouse/partner***						
None	Ref.					
Little	0.99 (0.56–1.77)	0.68 (0.25–1.83)	1.25 (0.78–2.00)	1.70 (0.94–3.08)	2.38 (1.23–4.63)	2.34 (1.49–3.67)
Moderate	0.95 (0.43–2.13)	0.92 (0.35–2.43)	1.30 (0.72–2.35)	2.13 (1.10–4.11)	4.06 (1.90–8.68)	3.32 (1.92–5.75)
Severe	1.02 (0.41–2.55)	0.37 (0.11–1.31)	1.20 (0.56–2.57)	1.48 (0.66–3.32)	3.47 (1.63–7.38)	6.19 (3.29–11.64)
						
***Number of Chronic Health Problems***						
One	Ref.					
More than one	2.00 (1.31–3.07)	1.77 (0.97–3.24)	1.41 (1.02–1.95)	3.34 (2.20–5.09)	2.25 (1.38–3.67)	1.56 (1.09–2.23)

Goodness of fit p value	0.65	0.03	0.14	0.72	0.39	0.90

As indicated in Table 3, female patients who declared that they had never been married and that they did not live with their partner were more likely to report a lower perception of their general health and higher levels of psychological distress than patients who were married and lived with their partner. Compared to patients who reported no conflict with their spouse/partner, patients who reported severe levels of conflict were more likely to have consulted their generalist and more likely to have a negative perception of their general health. Most notably, female patients who declared any level of conflict were more likely to report a lower perception of their mental health and higher levels of psychological distress. Finally, patients who reported more than one chronic health problem were more likely to have consulted a specialist, to have a lower perception of their general health and mental health.

**Table 3:** Female Patients. Odds ratios and 99% confidence intervals^† ^from logistic regression models^†† ^linking marital status, the perceived conflict with the spouse/partner, and the number of chronic health problems, to chronic illness management variables.

	**Dependent Variables**					
	
**Independent Variables**	Consulting a GP	Consulting a Specialist	Use of Info-Santé	General Health	Mental Health	Psychological Distress
***Marital Status***						
Married*	Ref.					
Common Law**	1.14 (0.79–1.66)	1.19 (0.73–1.94)	1.16 (0.87–1.55)	1.23 (0.80–1.88)	1.10 (0.72–1.68)	1.25 (0.89–1.75)
Wid/Div/Sep and do not live with their partner	1.33 (0.64–2.74)	1.17 (0.43–3.23)	1.21 (0.62–2.35)	2.12 (0.83–5.42)	1.71 (0.63–4.68)	1.53 (0.75–3.12)
Never married and do not live with their partner	1.17 (0.66–2.08)	0.71 (0.28–1.81)	0.85 (0.52–1.40)	2.45 (1.20–4.99)	1.93 (1.00–3.70)	2.04 (1.23–3.39)
						
***Conflict with spouse/partner***						
None	Ref.					
Little	1.18 (0.77–1.80)	1.14 (0.55–2.37)	1.34 (0.99–1.83)	1.24 (0.76–2.00)	2.25 (1.31–3.87)	1.81 (1.20–2.74)
Moderate	1.32 (0.84–2.09)	2.40 (1.38–4.18)	1.30 (0.85–1.99)	1.33 (0.75–2.34)	3.84 (2.29–6.46)	4.03 (2.71–5.99)
Severe	1.71 (1.07–2.74)	1.35 (0.69–2.63)	1.45 (0.94–2.25)	2.29 (1.36–3.88)	6.51 (3.76–11.28)	5.45 (3.40–8.75)
						
***Number of Chronic Health Problems***						
One	Ref.					
More than one	1,31 (0.97–1.78)	1.68 (1.08–2.61)	1.25 (0.99–1.57)	3.41 (2.21–5.25)	2.21 (1.45–3.38)	1.31 (1.00–1.73)

Goodness of fit p value	0.61	0.84	0.76	0.10	0.27	0.53

^† ^Each category of the independent variable is compared to the reference category; ^†† ^All models adjusted for age, sexe, education, employment status, and income.* married and living with their spouse; ** living with a partner and married/separated or widowed, or divorced, or never married

## Discussion

Linkages between characteristics of the couple relationship and illness management among chronically ill patients suggest that the patient's intimate couple context merits attention in studies of illness management. First, when the perceived conflict with the spouse/partner is examined in relation to measures of perceived health and psychological distress, strong associations are revealed between measures of self-perceived mental health and psychological distress and all levels of perceived conflict for both males and females. These findings take their meaning with studies that show that patients with higher emotional strain – such as in chronically conflicted relationship – report more depressive symptoms and a negative perception of their health [4,28].

Second, results of this study converge with previous research that indicates that gender is an important moderator of the association between structural and functional qualities of the couple relationship and health [4]. Two prominent gender differences stand out. The first is that marital status does not show any pattern of association with our measure of illness management for men, whereas women who declared that they were never married and did not live with their partner reported negative perceptions of their health and higher psychological distress than married women who lived with their spouse. The second gender difference is that perceived conflict in the couple seems to be associated with efforts at self-care (consulting a GP, consulting a specialist) for women but not for men. These results seem consistent with the view that, compared to men, women's self-representations, social information processing and self-regulation are mostly based on relational interdependence so that women's self-attributes, strivings and preferences are represented within the context of their couple relationships [4,29]. If this is the case, then women would be more sensitive and more responsive than men to the presence of conflict in the couple; their thoughts and feelings in the relationship would be partially regulated by and responsive to not only their behavior, but also that of their partner [4]. In addition, if we integrate this view with the one adopted in this study that marriage forms a social unit that may reflect a stronger commitment to the relationship, greater family organization, and sustained provision of support in the management of illness, then we should find that women who have not been married and do not live with their partner, may feel more alone in their struggle with the illness and report more negative perceptions of their health and higher levels of distress than married women who lived with their partner.

Further investigation of these findings, preferably with a longitudinal design, seems worthwhile. Previous research has for example shown that low family stress and high satisfaction with the couple relationship helps patients adapt better to the changes required by chronic illness [15,30] – and visits to the doctor are arguably part of this adaptive process. With the nuances that are due to gender differences in terms of self-representations and self-regulation, men and women may vary in terms of how their living arrangements and the quality of their intimate relationships affect their motivation to visit their doctor, or in terms of other variables associated with conflict in the couple. In depth analyses of the patients' living arrangements in terms of variations in the patterns of roles, family organization, marital functioning, spouse's specific behaviors and the level of marital satisfaction may help shed some light in this area [4,15].

Third, our study provides initial estimates of the use of telephone health line by patients with multiple chronic illnesses. Info-Santé, like other telephone advice nursing services, is intended to provide efficient responses to the users directing them to the appropriate care level and enhancing their self-care ability [31]. To our knowledge, the direct relations between couple conflict, multiple chronic problems, and the use of telephone health lines have not been systematically examined. We did not find any study that examined the influence of couple conflict on the use of telephone advice services. We found one study that reported a positive correlation between the number of health problems noted on the medical records and the number of advice calls in a random sample of HMO members [32]. Fewer than half of the patients had used telephone advice nursing services in a year, but no indication was reported of the number of health problems that these patients had. Further studies are required given the complexity of the factors that can influence the use of telephone help lines. Indeed, patients with multimorbidity may feel more urged to visit their doctors directly – given the complexity of their health problems – rather than call the nurses for advice, and among the factors that seem to encourage the calls are caller's perception of affective support, decisional control, and the perception of the nurses' competence [32].

Finally, the view of illness management as a system of interdependent variables seems to find a useful application in this study. In addition to strong associations of this system of variables with the number of chronic conditions, the analyses reveal gender-specific patterns of associations with the structural and functional features of the couple relationship that we have used in this research. These results highlight the need to examine multiple domains of family life separately by gender given that family characteristics have different influences on different aspects of illness management and may do so differently for males and females [6]. Other couple and family variables, within longitudinal designs and more elaborate models of illness management, may improve our understanding of the influence of the couple's relational context on what patients do to manage their illnesses and the results that they get.

The findings of this study are preliminary. In our exploration of couple conflict and illness management we have used a limited assessment of these constructs using the available variables of the Quebec Health Survey. Clearly, additional research with more structured and nuanced measures is required given both the complexity of family life and the variety of behaviors and strategies of self-care that chronically ill patients use [33,34]. Given the cross-sectional nature of this study, all of its results indicate statistical associations and we cannot derive any directional or cause to effect statement. Additional research should be longitudinal. Chronic illnesses often require continual adjustment of self-care over time to respond to the usual hassles of daily life and to the natural progression of the illnesses with their changing demands. Longitudinal designs would improve our understanding of how patient's illness management changes over time. Additional research should also use better measures of multimorbidity. In a recent study, we have found that a simple count of chronic conditions as a measure of multimorbidity can help uncover significant associations with quality of life outcomes [35-37]. However, the use of a multimorbidity index, the Cumulative Illness Rating Scale, revealed a stronger association with these outcomes than a simple count of chronic diseases [37]. In addition, although self-report of chronic disease diagnoses has been shown to agree with medical record diagnoses from 73% to 83% of the time [38], it can be argued that it is too subjective a measure of multimorbidity and that it can be confounded by the presence of depression [28]. Additional evaluations of specific clusters of diseases are also worthwhile given the variety of physiological and psychosocial pathways of influence on chronic illnesses.

## Conclusion

Despite its limitations, our analysis provide a useful preliminary assessment of the importance of living arrangements and the quality of the couple relationship in chronic illness management broadly conceived as a measure of the patient's efforts at self-care and an illness status indicator. Results of this study prod us to examine more closely, within longitudinal designs, the influence of living arrangements and the presence of conflict in the couple on chronic illness management as well as the modifying effect of gender on these associations.

## Competing interests

The author(s) declare that they have no competing interests.

## Authors' contributions

HS conceived this study, did the analyses, and wrote successive versions of the article. MF and CH reviewed the results, commented on successive drafts and on the study's findings. All authors approved the submitted versions of the paper.

## Pre-publication history

The pre-publication history for this paper can be accessed here:


